# Using Psychometric Testing Procedures for Scale Validity, Reliability, and Invariance Analysis: The PRETIE-Q Portuguese Version

**DOI:** 10.3390/ejihpe13070086

**Published:** 2023-06-23

**Authors:** Filipe Rodrigues, Pedro Morouço, Raul Antunes, Diogo Monteiro, Miguel Jacinto, Nuno Figueiredo, Filipe Santos, Vasco Bastos, Diogo Teixeira

**Affiliations:** 1 ESECS—Polytechnic of Leiria, 2411-901 Leiria, Portugal; filipe.rodrigues@ipleiria.pt (F.R.); raul.antunes@ipleiria.pt (R.A.); diogo.monteiro@ipleiria.pt (D.M.); miguel.s.jacinto@ipleiria.pt (M.J.); 1200180@my.ipleiria.pt (N.F.); 2 Life Quality Research Center (CIEQV), 2040-413 Leiria, Portugal; 3 Center for Innovative Care and Health Technology (CiTechcare), 2410-541 Leiria, Portugal; 4 Research Center in Sport Sciences, Health Sciences and Human Development (CIDESD), 5001-801 Vila Real, Portugal; 5 Faculty of Physical Education and Sport, Lusófona University, 1749-024 Lisbon, Portugal; a22212466@alunos.ulht.pt (F.S.); p7223@ulusofona.pt (V.B.); diogo.teixeira@ulusofona.pt (D.T.); 6 Research Center in Sport, Physical Education and Exercise and Health (CIDEFES), 1749-024 Lisbon, Portugal

**Keywords:** psychometrics, factor analysis, questionnaire, exercise

## Abstract

This study investigated the psychometric nature of preference for and tolerance of exercise intensity in physical activity. It initially re-examined the Preference for and Tolerance of the Intensity of Exercise Questionnaire (PRETIE-Q) among Portuguese exercisers, looking at its applicability to different exercise activities and exercise experiences. Then, to investigate the applicability of the measure in different groups, its invariance was examined. The sample consisted of 1117 participants (528 male, 589 female) aged 18–81 years old (Mage = 36.81, SD = 11.89). All participants reported at baseline that they were exercising, on average, 3.93 days (SD = 1.36) per week. The exploratory structural equation modeling (ESEM) displayed the best fit. The ESEM did show invariance when tested for multigroup analysis. The conclusion of this research is that the ESEM demonstrated the best fit, displaying invariance in multigroup analysis. Furthermore, when assessing preference and tolerance in various exercise modalities, the PRETIE-Q should be primarily used as a multidimensional instrument due to the differential recognition of preference and tolerance in seemingly similar physical activity circumstances, highlighting the importance of employing context-verified measures to evaluate exercise-intensity preference and tolerance based on sample characteristics or real-time context.

## 1. Introduction

Exploratory factor analysis (EFA) is a widely used statistical method in behavioral and social sciences. This statistical technique is used in the field of psychometrics to find underlying patterns in a set of variables. The goal of EFA is to identify the underlying structure of a set of observed variables and to simplify the data by decreasing the number of variables to a smaller set of uncorrelated factors. However, while EFA is a suitable place to start, it has several limits. For example, it lacks a measure of model fit, making it difficult to assess the quality of the results. Furthermore, it lacks evidence of structural validity, which is a measure of how effectively a test or questionnaire assesses what it is supposed to measure. Confirmatory factor analysis (CFA), a more systematic technique for data analysis, may be used by researchers to address these constraints. CFA allows researchers to test particular hypotheses about dataset factor structure and offers a measure of model fit that may be used to make model change decisions. Furthermore, CFA gives evidence of structural validity, which can be utilized to improve test or questionnaire validity [1]. The differences between these two-factor analytics techniques, as Marsh et al. [1] point out, is that: (a) EFA is an exploratory strategy for identifying patterns in data, whereas CFA is a hypothesis-testing approach for testing specific assumptions about a dataset’s factor structure; (b) EFA is the process of reducing a large number of variables into a smaller set of uncorrelated variables that can be utilized to describe the relationships between the original variables, whereas CFA is used to evaluate certain hypotheses about the dataset factor structure, which is predetermined; and (c) EFA is the process of condensing a large number of variables into a smaller set of uncorrelated variables that can be used to characterize the original variables’ relationships. Because CFA is used to test hypotheses about the factor structure of a dataset, the factor structure is predetermined. As a result, EFA cannot make use of the recent developments in latent modeling associated with CFA [2].

CFA has been the go-to technique for assessing factor structures when it comes to scale development, refinement, and validation [3]. Its primary advantage in psychological assessment is the ability to directly assess the relationship between later factors [1]. Based on assumptions proposed by Howard et al. [3], instruments that fail to meet adequate fit adjustment requirements in CFA are of little use. As a result, the independent cluster model in CFA (ICM-CFA) has been developed, which provides a distinct way to understand the structure of a set of variables. The ICM-CFA technique entails grouping variables that are thought to be connected to one another based on their correlation patterns. These clusters are then represented as separate variables in a CFA model, which gives a model fit measure as well as evidence of structural validity. This method allows for the discovery of new factors that may then be investigated further using classic EFA methodologies. However, in many cases, this method has proved unduly restricted, failing to provide clear support for instruments that appeared to be well established in EFA tests [4]. Given the benefits of EFA and CFA, in this study, exploratory structural equation modeling (ESEM) will be used as it has demonstrated advantages in factor analysis in social sciences research [1,5].

ESEM is a statistical method developed as an alternative to standard CFA and EFA. The ESEM technique, which was created by Bengt Múthen and colleagues [1,5], has been widely used in the fields of psychology and other social sciences. The primary premise of ESEM is that the data being examined have a complicated structure that cannot be effectively described by a simple factor model. Higher-order latent variables and non-linear correlations between variables are common characteristics of this structure. The ESEM technique models these complicated relationships in a more flexible and realistic manner than standard CFA or EFA approaches. As a result, ESEM integrates assets from each technique into a single analytical evaluation in which variables with cross-loadings coexist with ICM-CFA assumptions [1,5]. The goal of rotating the loading matrix in ESEM is frequently to simplify the structure of the model and make it easier to interpret. Researchers can adjust the factor structure of the model by rotating the matrix, resulting in changes in both the measurement and structural coefficients. Measurement coefficients represent the strength of the relationship between the indicators and the latent constructs they are measuring. They are also known as factor loadings or factor load coefficients. Structural coefficients, on the other hand, refer to the parameters that describe the relationships between the latent constructs themselves. These coefficients indicate the strength and direction of the causal relationships between the latent constructs. They are also known as path coefficients [5]. This can be helpful in determining the most meaningful and parsimonious factor structure for the data. As a result, Asparouhov and Muthén [5] characterize ESEM as providing standard errors for all rotational parameters. In ESEM, overall model fit tests are obtained to assess the goodness-of-fit of the model to the data. These fit indices provide information about how well the model represents the underlying relationships between the observed variables and latent variables. This factor analysis provides a versatile and complete way to model the interrelationships between variables, including the capacity to model complicated structures and non-linear relationships. It does not require further study with structural exploratory modeling (SEM) because ESEM has all of the SEM qualities [5]. ESEM may be an alternative to EFA, CFA, and SEM as separate statistical techniques in this regard.

The current study also looked at the increasing usage of bifactor modeling and its application in psychometric testing [2]. Bifactor modeling is a type of psychometric modeling that is commonly used in CFA and SEM frameworks. In this type of bifactor modeling, a latent variable is hypothesized to be responsible for the shared variance among a set of observed variables, while each observed variable is also hypothesized to have a unique association with the latent variable. Thus, a bifactor model is a structural equation model that combines a general factor and specific factors, each of which accounts for different features of the data. This method is especially beneficial when numerous factors are anticipated to contribute to the observed variables, and a general factor is thought to represent a broad underlying construct that is shared by all of the variables. Specifically, a bifactor CFA model loads each item on both a general and a specialized factor. As a result, when studying the model, the general factor has direct loadings rather than indirect loadings through the specialized factor, as in hierarchical models [6]. According to Byrne, whereas hierarchical models are widespread in the literature, bifactor models are not [7]. This could be owing to very restrictive implicit norms in practice [2]. Nonetheless, a bifactor ESEM model can provide a more flexible way of determining if the presence of a global factor better captures the underlying data than the specification of two or more related but independent factors.

Bifactor exploratory structural equation modeling (bifactor ESEM) is a new advancement in SEM that combines the benefits of bifactor models and ESEM [2]. Bifactor ESEM extends the standard bifactor model by including ESEM’s flexible, exploratory methodology, allowing for a more extensive study of variable connections [1,2,5]. First, the general and specific factor loadings, as well as the measurement errors, which represent the error associated with each observed variable, can all be estimated using bifactor ESEM models. This information can then be utilized to improve the model’s fit to the data. Second, this approach provides a thorough and nuanced knowledge of the variables’ relationships. The model’s bifactor structure allows researchers to distinguish between the contributions of the general factor and specific factors to the observed variables, offering a more detailed understanding of the variables’ interactions. Last, bifactor ESEM models handle the issue of non-target factors by allowing estimation of general and specific factor loadings as well as measurement error. These data can then be used to identify and account for the effect of non-target variables on the observed variables [1,2,5]. The bifactor ESEM approach may be more comprehensive and versatile than EFA, CFA, or SEM alone, and ESEM can be performed while using a confirmatory bifactor target rotation method [2,8].

### 1.1. The Case of the Preference for and Tolerance of the Intensity of Exercise Questionnaire (PRETIE-Q)

The pursuit of a pleasurable and enjoyable experience has been proposed as an important contributor to exercise intention, persistence, and adherence to physical activity programs [9,10,11,12]. Exercise intensity is one of the characteristics that have a strong relationship with pleasure and enjoyment [13]. Several studies have found that increasing exercise intensity has a considerable impact on affective states, with higher intensities being associated with decreased pleasure or increased displeasure [10,12,14,15].

This intensity–pleasure relationship is highly variable among individuals, particularly at mid-range intensities (not too low, not too high). Individuals differ substantially in the intensity of exercise that they enjoy and can tolerate and hence in the degree to which exercise can elicit an appropriate affective response, as stated by Ekkekakis et al. [16]. Additionally, affective responses to exercise sessions are related to future intentions toward physical activity [10]. The sensory modulation of exercise-induced intensity-related stimulation is assumed to be linked to the individual difference variables of (a) exercise-intensity preference and (b) exercise-intensity tolerance. As a result, effective measures that assess and clearly differentiate preference and tolerance for exercise intensity and its applicability based on exercise type (e.g., resistance training vs. aerobic training), as described by existing literature, are required. [17,18].

The PRETIE-Q, created by Ekkekakis et al. [19], is a 16-item questionnaire designed to assess the traits of preference (i.e., inclination to choose a specific level of exercise intensity) and tolerance (i.e., inclination to continue exercising at an imposed level of intensity even when the activity is unpleasant/uncomfortable). With eight items each, the scale examines two dimensions (preference and tolerance), and respondents use a 5-point Likert scale ranging from “I strongly disagree” to “I totally agree” to respond to each item. Half of the Preference questions (i.e., 2, 4, 8, 12) and half of the Tolerance items (i.e., 1, 3, 9, 13) assess LOW Preference and LOW Tolerance. These items must be scored backward. These items are reverse-scored so that higher responses now reflect a lower preference and tolerance score and vice versa.

After the first development and validation study for the PRETIE-Q [19], the following studies were published in an attempt to validate, complete, and broaden the applicability of this measure: (1) psychometric testing of the preference scale [19]; (2) psychometric testing of the tolerance scale [16]; (3) cross-validation in college women [16]; (4) preliminary testing of the Brazilian–Portuguese version [20]; (5) preliminary testing of the Portuguese version [21]; and (6) initial testing of the Chinese version [22]. In conclusion, the questionnaire’s creation and further testing demonstrated psychometric qualities in a variety of cultures and contexts. Furthermore, the Portuguese (10 items; 5 per construct; 10 items represent low preference/tolerance) and the Chinese (8 items; 4 per construct; 4 items represent low preference/tolerance) versions presented a distinct final set of items compared to the original version, which may be crucial for the questionnaire’s quality comprehension. Patterson et al. [23] highlighted this while claiming that the questionnaire would benefit from redesigned and reduced scales, a problem that further psychometric testing could address.

### 1.2. Current Research

This study aimed to apply bifactor ESEM procedures to examine the validity, reliability, and invariance of the PRETIE-Q Portuguese version in different exercise modalities and exercise experiences. In addition, it also sought to analyze correlational validity with enjoyment, exercise intentions, and exercise frequency. According to the literature, preference, and tolerance of exercise intensity are assumed to be discriminant in their influence on affective responses [19], and several studies have supported this view (e.g., [24]). Although Teixeira et al. [21] validated the PRETIE-Q Portuguese version, their scale was not examined for different exercise types and according to exercise experience. In addition, the mentioned study did not measure invariance between exercise experience. However, both analyses are important to support instrument validity [25]. Cid et al. [26] proposed that measurement research should be performed in specific cultural contexts and that scales validated in one context should not be utilized in another unless thorough cross-cultural validation has occurred. In addition, Ekkekakis [27] advocated for the development and deployment of appropriate questionnaires tailored to each individual scenario, and this idea was also supported by Cid et al. [26]. Teixeira et al. [24] emphasized this point when they applied the Portuguese version to 445 Portuguese individuals. Furthermore, given the distinction between preference and tolerance constructs, Teixeira et al. [24] highlighted the need for additional data in different scenarios to demonstrate the universal use of this comprehensive instrument for assessing both facets of intensity-preference and intensity-tolerance traits in all sorts of exercise modalities.

The primary objective of this study was to evaluate the psychometric properties of the PRE-TIE-Q Portuguese version in a large sample of Portuguese adults who engage in diverse exercise activities. To achieve this, we employed confirmatory factor analysis (CFA) and exploratory structural equation modeling (ESEM), along with bifactor modeling methodologies. The initial focus was on investigating the factor structure of the PRETIE-Q within the exercise context. Notably, to our knowledge, no previous study has directly compared these techniques to determine the strength of a general factor, specifically in the exercise domain. The bifactor analysis in this research aimed to determine whether the items assessing exercise-intensity preference and tolerance load onto a single global factor while also allowing for the estimation of specialized factors for each dimension of exercise intensity. By examining these specific factors, we sought to gain a comprehensive understanding of the underlying constructs associated with exercise intensity.

The secondary objective of the study was to explore the context invariance between exercise activities and exercise experience. By conducting multigroup invariance tests, we aimed to determine whether observed group differences in the PRE-TIE-Q scores reflected genuine variations in latent factors or were influenced by other variables. This assessment of context invariance is crucial for establishing the validity of the instrument and its applicability across different exercise modalities and levels of exercise experience [25]. To test these objectives, we formulated the following hypotheses: (a) the PRE-TIE-Q would demonstrate factor validity across all models, affirming its reliability as a robust instrument for measuring both preference for and tolerance of exercise intensity as two correlated factors; and (b) measurement invariance would be present across all exercise modalities and exercise experience levels, indicating that the instrument performs consistently across different contexts. By addressing these research goals and testing the formulated hypotheses, this study aimed to enhance our understanding of the psychometric properties of the PRE-TIE-Q Portuguese version and its suitability for assessing exercise-intensity preference and tolerance in a diverse range of exercise contexts.

## 2. Methods

### 2.1. Participants

The a priori sampling calculator for factor analysis [28] was used to calculate the minimum sample size required for this study to be valid and reliable. The following inputs were used considering existing evidence [20]: number of observed variables = 8; probability level = 0.05; anticipated desired statistical power = 0.80; effect size = 0.15 (small effect); and number of latent variables = 2. The results suggested that the minimum number of participants was 411 for the results to be valid and reliable.

The sample consisted of 1117 participants (528 male, 589 female) aged 18–81 years old (Mage = 36.81, SD = 11.89). All participants reported at baseline that they were exercising, on average, 3.93 days (SD = 1.36) per week. Participants were actively engaged in fitness group classes (n = 552) or aerobic and/or resistance training (n = 563). Regarding exercise experience, it ranged between 0 and 600 (M = 12.99; SD = 34.25) months. Individuals were grouped based on exercise experience considering the 6-month cutoff, as used in previous literature [11], specifically: 493 (44.1%) had less than 6 months of exercise experience, and 624 (55.9%) had more or equal to 6 months of exercise experience. Concerning body mass index, participants self-reported height and weight, indicating to be normal weight (65.4%), overweight (21.8%), or obese (12.8%). For inclusion, those who met the following inclusion criteria were considered: (i) aged 18 years old or older; (ii) provide informed consent to participate; and (iii) be an active gym or fitness center member.

### 2.2. Procedures

This study was conducted with ethical institutional approval (CE/IPLEIRIA/35/2021). Several gyms and fitness centers were contacted after receiving ethical institutional approval. Because the researchers had access to potential volunteers, they employed a convenience sampling strategy to collect data. The club managers were each given an explanation of the objectives and data collection techniques. Following club management clearance, potential participants were approached via an internal e-mail list and requested to engage voluntarily in this study. All participants were informed about the study’s objectives, and signed informed consent was collected individually. A self-administered online form questionnaire was used to collect data from participants. The questionnaires were completed in less than 10 min on average.

### 2.3. Instruments

The PRETIE-Q Portuguese version [21] consists of ten items representing the two scales that correspond to the intensity-preference (e.g., “The faster and harder the workout, the more pleasant I feel”) and intensity-tolerance (e.g., “Feeling tired during exercise is my signal to slow down or stop”) traits. Half of the items on each scale have inverted scores. Prior to data analysis, all negatively oriented items were reverse-scored to align with the polarity of the positively oriented items. This procedure was performed in IBM SPSS Statistics version 27. The stem asks respondents to rate what best represents their beliefs and feelings when exercising on a 5-point bipolar Likert scale, ranging from 1 (“Totally Disagree”) to 5 (“Totally Agree”).

The Physical Activity Enjoyment Scale Portuguese version [29] was also used in the present study. This 4-item short version (e.g., “It is very stimulating”) scale assesses the level of agreement on enjoyment when exercising. The assessment of perceived enjoyment is reflected in the responses given on a 7-point bipolar Likert scale ranging from 1 (“Totally disagree”) to 7 (“Totally agree”). The score is computed by taking the average of the data from each item.

The intention was assessed using a Portuguese validated scale [30] grounded on the theory of planned behavior to measure intention toward exercise in the future. Three items evaluate the intention to continue exercising (e.g., “I will continue to exercise in the next 6 months as I currently do”) using a 7-point scale anchored from 1 (“Absolutely not”) to 7 (“Absolutely yes”).

Participants were asked to report their weekly exercise frequency during the previous week (question: “How many days per week do you believe you exercised over the last week?”). In the past, a single-item measure of exercise frequency was regarded as a valid and trustworthy indicator of exercise practice. [31].

### 2.4. Statistical Analysis

#### 2.4.1. Factor Analysis

All analyses were carried out using the Mplus version 7.4 software with a robust maximum likelihood (MLR) estimator [32]. The authors of the study conducted an initial analysis at the level of item responses to evaluate the distributional properties of the data. They observed that some items 6, 7, and 10 displayed skewed (scores > 7) distributions that could indicate departures from normality. Researchers opt to use the MLR estimator for several reasons. The MLR estimator is robust to violations of normality assumptions and can provide accurate parameter estimates in the presence of non-normal data [33]. Second, categorical data often exhibit non-constant item variance, where the variance of responses may vary across different items. Third, the MLR estimator can account for non-constant item variance, which can lead to more accurate and robust parameter estimates [34]. Last, the MLR estimator in Mplus can handle missing data using robust techniques, which provide robust parameter estimates even in the presence of missing data.

To address the small amount of missing data at the item level across all instruments (missing data mean = 3%), full information maximum likelihood (MLR) estimation was used for all data analyses, which assumes data are missing at random (MAR). Previous theoretical [19] and empirical [21,22,35,36] research has supported modeling based on two correlated specific factors. As a result, two-factor structure configurations were examined, namely unidimensional and two correlated factors via CFA and ESEM and a bifactor via CFA and SEM (see Supplementary Materials of the Mplus syntaxes).

In CFA models, items were only allowed to load on their predefined factors, limiting cross-loadings on undesirable factors. Furthermore, both elements were permitted to coexist. Oblique target rotations were used in the ESEM model. In other words, factors were defined similarly to CFA models, but cross-loadings were free to be estimated while assuming they were close to zero. Items were input into their predetermined specific factors and a global factor in bifactor CFA models, and all specific factors were permitted to associate freely. The bifactor ESEM model was employed in this study, which is identical to the bifactor CFA model except that all cross-loadings for the various factors were estimated freely using oblique rotations.

Chi-square statistics are frequently used to assess the fit of measurement models. However, due to their sensitivity to sample size and model specifications [35], the current study assessed model adequacy using a variety of common goodness-of-fit indices, namely the Tucker–Lewis index (TLI), comparative fit index (CFI), standardized root mean residual (SRMR), and root mean square error of approximation (RMSEA) and its respective confidence interval at 90% (CI 90%). CFI and TLI values of ≥0.90 are generally regarded as adequate [7,37,38], and SRMR and RMSEA values of ≤0.08 suggest a reasonable fit to the data [1,36]. Marsh et al. [1] point out that these are only guidelines because ESEM and bifactor models are rarely utilized, leaving the efficiency of these indices, and proposed cutoff scores for further investigations. For bifactor model processes, the conventional goodness-of-fit indicators utilized in CFA and SEM model specifications were investigated.

For the evaluation of the standardized factor loadings, a value equal to or greater than 0.50 was considered acceptable. The interpretation of a standard factor loading of 0.50 means that 25% of the variance in the observed item can be explained by the latent factor, controlling for all other latent factors or covariates in the model [39]. The omega coefficient [40] was estimated for the subscale scores to test internal consistency in the two-correlated model solutions, with values of 0.70 being considered satisfactory [41]. If the bifactor model specifications are found to be acceptable, the omega composite reliability coefficient [42] for bifactor models is determined, as it takes into account the strength of the correlation between items and specific factors, as well as item-specific measurement error [43,44]. The average variance extracted (AVE) approach was used to test convergent validity. The constructs are identified as separate when the square root of each AVE value is greater than the correlation between the two constructs and the AVE for each construct is greater than 0.50 [45].

#### 2.4.2. Multigroup Analysis

To study measurement invariance across exercise type and exercise experience, the best model fit from the factor structure analysis was originally investigated in each group independently. Following then, various levels of measurement invariance were measured in accordance with the suggestions of several authors [2,46]. Each of the four measurement invariance levels builds on the preceding level by imposing more equality restrictions on the model parameters, resulting in stronger types of invariances. The parameters known to be invariant from previous levels are lowered when each new set of parameters is reviewed. As a result, determining measurement invariance is essentially a series of ever more constrained hypothesis testing. The following levels were considered: configural invariance (i.e., factor structure is the same between groups; same items are associated with the same factors); weak factorial invariance (i.e., factor structure and factor loadings are equal between groups); strong invariance (i.e., item factor structure, factor loadings, and item thresholds are equal between groups); and strict factorial invariance (i.e., item factor structure, factor loadings, and item thresholds are equal between groups (i.e., item factor structure, factor loadings, item thresholds, and item residuals are equal between groups).

To make model comparisons, the following assumptions were used: (a) differences in CFI and TLI should be ≤0.01 for configural invariance [4], supplemented by a change of ≤0.015 in RMSEA or a change of ≤0.030 in SRMR would indicate invariance; (b) for weak factorial, strong and strict factorial invariance, a change of ≤0.010 in CFI, supplemented by a change of ≤0.015 in RMSEA or a change of ≤0.010 in SRMR would indicate acceptable criteria for invariance [25]. It is important to note that these are guidelines because multigroup analysis employing bifactor CFA or ESEM is unusual [1].

#### 2.4.3. Correlational Analysis

SEM with latent variables was performed for correlational analysis between preference for and tolerance of exercise intensity and subjective vitality, using the same model acceptability guidelines suggested by various authors [7,39]. A correlational validity analysis was carried out, considering preference for and tolerance of exercise intensity as independent variables and enjoyment, exercise intentions, and exercise frequency as dependent variables. The direct effects of each exercise-intensity trait were examined using standardized coefficients and their respective 95% confidence intervals (CI95%). If the CI95% did not include zero, the regression path was considered significant [47].

## 3. Results

Fit indices of the five models for the PRETIE-Q Portuguese version’s psychometric proprieties are exhibited in Table 1. The two correlated factors of CFA and the bifactor model specifications did not achieve an acceptable level of fit to the data. However, the two correlated factors of the ESEM model solution achieved a suitable fit (CFI and TLI > 0.90; and RMSEA < 0.08). In this regard, this study moved on to examining the factor loadings and convergent analysis.

The factor loadings from the two-correlated-factors ESEM models are shown in Table 2. All items loaded to targeted factors with values larger than 0.50. However, cross-loadings were discovered in the ESEM model. Cross-loadings showed variations of less than 0.15, except for Item 6, indicating different causes. Thus, items were retained in the respective factor. With respect to the composite reliability coefficients, the results showed scores above acceptable in both constructs. The correlation between latent factors was positive and significant (r = 0.606; *p* < 0.001). Convergent analysis showed that preference (AVE = 0.505) displayed acceptable scores, but tolerance did not (AVE = 0.372). However, discriminant validity was achieved since the squared correlation of factors (*r*^2^ = 0.364) was below AVE scores, suggesting that both factors are distinct from each other.

The ESEM was used to test measurement invariance between groups since it provided a better fit to the data compared to the other models (see Table 1). The measurement model fits the data well in each group independently. That is, the two-correlated-factor ESEM displayed acceptable fit in resistance and cardio training, fitness group classes, <6-month experience, and ≥6 months experience subsamples. The measurement invariance results show that the multigroup analyses provide evidence that strong invariance was tenable across both exercise type and exercise experience groupings (see Table 3). As shown in Table 3, multigroup analysis between contexts did achieve levels of invariance (ΔCFI and ΔTLI > 0.01; ΔRMSEA > 0.015) except for strict.

Exercise-intensity preference and tolerance, enjoyment, exercise intentions, and exercise frequency were examined in the SEM model. The specified SEM model considering exercise-intensity traits and all dependent variables fit the data reasonably (χ2 = 1051.702, df = 129, CFI = 0.925, TLI = 0.911, SRMR = 0.076, RMSEA = 0.080). The results showed that preference (β = 0.43; *p* < 0.05) and tolerance (β = 0.30; *p* < 0.05) were positively and significantly correlated with enjoyment. The results also showed that preference (β = 0.18; *p* < 0.05) and tolerance (β = 0.18; *p* < 0.05) were positively and significantly correlated with exercise intention. The results showed that preference (β = 0.23; *p* < 0.05) and tolerance (β = 0.25; *p* < 0.05) were positively and significantly correlated with exercise frequency. The findings of this study provided further support for the earlier research using different versions of the PRETIE-Q.

## 4. Discussion

The current study investigated the multidimensionality of the PRETIE-Q [16,21] among participants involved in various physical activities and its relationship to the preference for and tolerance of exercise intensity. Second, it also investigated PRETIE-Q applicability across Portuguese exercisers in terms of its ability to support preference and tolerance use according to the exercise type and exercise experience. This research was conducted using a recently established bifactor ESEM approach that integrates EFA, CFA, and SEM to examine the multidimensionality of exercise-intensity traits in a more complete manner. Overall, the findings supported the applicability of the PRETIE-Q within the physical activity context, using two factors as the primary measure.

The present study’s findings can help academics obtain a better understanding of psychometric research, which is especially essential in Portuguese exercise research, where research in sports and exercise psychology is thriving [26]. However, no previous work had explored the PRETIE-Q psychometric qualities in an exercise population using bifactor model specifications nor assessed the scale across various groups in the context of exercise.

### 4.1. Factor Structure

The current research’s initial step was to evaluate the factor structure of the PRETIE-Q, assessing multiple distinct models. According to the recommendations of previous literature [7,38], this study started with the unidimensional model. However, it did not fit the data well. The two correlated factors from CFA also did not fit the data well. Still, the two-correlated-factors ESEM displayed an acceptable fit to the data. While only the ESEM specification was indicative of an acceptable fit, the results are similar to those displayed in previous studies using this measure [16,19,21,22].

Items loaded in the ESEM model according to specified factors, with values greater than 0.50 and explaining at least 25% of variation [39]. Although numerous items showed cross-loadings in the ESEM model, only cross-loadings in Item 6 presented differences higher than 0.15. Looking at the description of the item (“*I would rather have a short, intense workout than a long, low intensity workout*”), it seems that participants have some difficulties in assessing the item as preference or tolerance-oriented. Nevertheless, the item was retained in the theoretically proposed factor as a means to maintain a parsimonious model. In addition, its removal or its redefined loading to the tolerance factor did not increase model fit. Nevertheless, future studies should revise item meaning to examine if the item measures what it is intended to measure. Overall, nine items significantly loaded the corresponding factor, showing an appropriate indication of different factors, which is in line with the findings of prior authors [7,39]. Teixeira et al. [21] found similar results, suggesting that Item 10, while displaying low factor loading, should be retained as it loads significantly on the tolerance factor. Looking at composite reliability coefficients, the present study found that scores in the two-correlated-factors SEM model were above acceptable (see Table 3). When assessing preference for and tolerance of exercise intensity, several studies [19,21,22] found similar results for composite reliability coefficients using CFA analyses. As a result, the two-correlated-factors ESEM shows consistent results with prior PRETIE-Q measurement investigations in exercisers and active individuals.

This study was the first attempt to investigate the psychometric proprieties of the PRETIE-Q using bifactor specifications. Regarding bifactor models, both CFA and ESEM did not fit the data well. When studying the factor structure of an exercise-intensity trait measure based on hedonic assumptions, it appears to be robust to assume preference and tolerance as distinct measures, showing that there is no theoretical nor statistical indication of a global factor of exercise intensity [21]. The current findings demonstrated that the PRETIE-Q questions better reflected specific intensity-preference and intensity-tolerance constructs rather than a global representation.

### 4.2. Multigroup Analysis

The assessment of invariance was a crucial and critical feature of the current study and one that has been under-researched using this measure. This study evaluated participants from two exercise types, including resistance and cardio training, as well as fitness group classes and exercise experience, to verify that comparisons of the PRETIE-Q between groups were reliable. The study found that the two-correlated-factors ESEM measurement model fit groups reasonably well. Specifically, the results demonstrated the attainment of configural invariance, suggesting that the factor structure of the PRETIE-Q was the same for both groups (see Table 3). Weak factorial invariance was also achieved, resulting in equal factor loadings between groups. Furthermore, the construction of strong and rigorous invariance demonstrated that item thresholds and item residuals were equal between groups [2].

The models failed to provide invariance at the strict level. This level of invariance refers to a higher level of measurement equivalence or invariance across different groups being compared, and it implies that the factor loadings, intercepts, and error variances of the observed variables in the measurement model are constrained to be equal across groups, and the latent variables are assumed to have the same meaning and measurement properties across groups. That is, the model failed to require that the factor loadings, as well as the intercepts and residuals, would be identical across groups. These results could be attributed to the sampling variability since exercisers can practice both resistance and cardio training or fitness classes according to their daily preference. While participants were asked to report the most practiced type of fitness activity, there could be some challenges in differentiating types within the context. The results can also be attributed to the exercise experience since it is measured as a continuous variable. As participants were grouped according to the 6-month exercise threshold, their perception of preference and tolerance can vary within the same group (e.g., one individual with 10 years and another with only 1 year of exercise experience). Forthcoming studies should explore these limitations. The accomplishments of invariance at the configural, weak, and strong levels confirm the findings of Teixeira et al. [21], also conducting multigroup analysis at the longitudinal level and reacting to their advice to further investigate invariance of the PRETIE-Q. However, the lack of strict invariance shows the need for further psychometric analysis of the PRETIE-Q in the exercise context. It is important to carefully examine and address these potential sources of lack of strict invariance when conducting multigroup factor analysis to ensure that valid and meaningful comparisons can be made across different groups of exercisers. Nevertheless, the PRETIE-Q appears to be a valid tool for assessing exercise-intensity traits in a sample engaging in different exercise types and with different exercise experiences since the measurement model displayed acceptable fit in each group (see Table 1) in the context of gym and health club activities.

### 4.3. Correlational Analysis

The SEM model provided a suitable fit for the data. Significant associations were found between factors under analysis (i.e., enjoyment and intention), as hypothesized earlier. Thus, the SEM model provided evidence for the predictive validity analysis between preference and tolerance, and enjoyment, exercise intentions, and exercise frequency. According to the results, a higher perceived preference for and tolerance of exercise intensity indicates a higher perception of enjoyment, exercise intention, and exercise frequency. This can be partially explained by previous research [24,33], in which exercisers who preferred and tolerated higher exercise intensity had greater perceived enjoyment *when* the training plan was congruent with their preferences. Additionally, having higher preferences in these constructs may facilitate an improved affective response [10,12], a known predictor of exercise adherence, given that it may act as a buffer for higher and sometimes misadjusted exercise intensities [24]. These conditions may lead to greater intentions to repeat the behavior, as the behavior is perceived as enjoyable [29,30]. Therefore, it is expected that training sessions should be exercise oriented based on preference and tolerance [21]. While the focuses were the psychometric tests of the PRETIE-Q and how latent factors can be explored using different psychometric procedures, the model supports theoretical and statistical evidence of the variables under analysis.

### 4.4. Limitations and Directions for Further Research

Some limitations of the current study should be acknowledged when evaluating the findings. This was the first study to provide support for the validity of the PRETIE-Q using more contemporary psychometric testing procedures. More empirical studies using the PRETIE-Q in the exercise setting are desired, as is a replication of previous measurement findings in different cultural situations to determine generalizability. In the case of the Portuguese version of this instrument, future efforts should be made to test all the items that were removed in the original validation process, an issue that the current study did not address [21]. Given that the PRETIE-Q consists of two factors, each made up of half positively oriented and half negatively oriented, and future studies could consider other models that allowed for two correlated general factors (i.e., preference and tolerance) with two specific factors (i.e., positively oriented items and negatively oriented specific factors). This analysis could also consider a general factor with four specific factors, and by doing so, the model would conflate the orientation of the items and the content of each substantive dimension. Several academics have stressed the importance of construct validation as a continual process [27,48]. It is also worth mentioning that because this is a cross-sectional study, drawing causal conclusions is impossible.

Exercisers are known to have diverse levels of experience and engage in more than one exercise type, and therefore, heterogeneity should be expected. Despite the relatively large sample size, future research should recruit more diverse samples from the exerciser population. Age and other sociodemographic modifiers, for example, may influence preference for and tolerance of exercise intensity. Nonetheless, it is worth repeating that, as previously reported, the PRETIE-Q was consistent across sex [21].

Additionally, the constraints of self-reporting measures must also be recognized. Accurate self-report relies not only on honesty and an absence of social desirability/bias but also on a level of participant self-awareness. Some exercisers may not regard themselves as high in preference for and tolerance of exercise intensity in the same way [35]. Future research may take this into account while grouping individuals based on the degree of intensity (low, moderate, vigorous). Finally, while the findings showed significant correlations between these exercise-intensity traits, enjoyment, exercise intentions, and exercise frequency, there are other cognitive or affective factors that could emerge as potential consequences. For example, Bastos et al. [17] discovered that intensity traits positively predict positive affective valence and activation. As a result, future study into the relationships between affect factors in exercisers, an under-researched group, is critical. Fitness professionals establish the motivational climate and effect for exercisers to maintain their engagement and to benefit from exercise. Therefore, it is crucial that a study program that studies the motivations of their leaders is developed to influence policy and practice in the exercise sector.

## 5. Conclusions

The findings of this investigation confirmed the PRETIE-Q factor structure and adaption to Portuguese exercisers while applying contemporary statistical tests beyond traditional confirmatory factor analyses. The exercise-intensity traits were found as different constructs that represented hedonic assumptions once more. Furthermore, the PRETIE-Q demonstrated itself to be a valid and reliable 10-item measure to assess their preference for and tolerance of exercise intensity in the gym and health club context. The findings support the application of the PRETIE-Q in exercisers, providing additional proof of the factor structure in a physical activity environment, as previously shown in other studies [21,24]. The PRETIE-Q instrument holds significant potential to contribute to research on hedonic assumptions in the exercise context, specifically in analyzing the impact of exercise-intensity traits on exercise adherence among individuals engaging in physical activity. This current study serves to augment the existing body of evidence supporting the use of the PRETIE-Q in exercise research, enabling researchers to obtain reliable estimations of preference and tolerance for exercise intensity, thus facilitating appropriate exercise prescription. While the measurement model did not demonstrate strict invariance across groups, the findings of this study provide an impetus for future research to explore the stability and validity of the PRETIE-Q factor structure in diverse physical activity settings and different populations. It is important to note that scale assessment is a complex and evolving process, and it should not be perceived as a rigid or definitive technique. Consequently, this work lays the groundwork for future evaluations of measurement instruments to employ more sophisticated statistical approaches, thus showcasing their strengths and limitations when defining items and targeted constructs.

### Practical Implications

The practical implications of this study are two-fold. Firstly, researchers can confidently employ the PRETIE-Q instrument as a reliable measure to assess exercise-intensity preference and tolerance in gym and health club settings, enabling them to gain insights into hedonic assumptions, tailor exercise prescriptions, and enhance exercise adherence among individuals engaged in physical activity. Secondly, exercise physiologists can benefit from utilizing the PRETIE-Q to obtain reliable estimations of individuals’ exercise-intensity preference and tolerance, allowing them to customize exercise prescriptions according to clients’ specific needs and preferences. By recognizing the complexity of scale assessment and considering the instrument’s demonstrated validity and reliability, exercise physiologists can effectively incorporate the PRETIE-Q into their practice, addressing hedonic assumptions associated with exercise intensity.

## Figures and Tables

**Table 1:** Model fit indices.

Model	χ^2^	df	CFI	TLI	SRMR	RMSEA	90% CI
Unidimensional	953.951 *	35	0.701	0.615	0.089	0.153	0.145, 162
Two-correlated-factor CFA	840.500 *	34	0.812	0.758	0.064	0.122	0.113, 0.130
Two-correlated-factor ESEM	219.335 *	26	0.937	0.911	0.033	0.080	0.072, 0.092
One bifactor and two-correlated CFA	DNC						
One bifactor and two-correlated ESEM	116.568 *	18	0.812	0.799	0.024	0.070	0.058, 0.082
Resistance and cardio training	201.970 *	26	0.927	0.911	0.055	0.075	0.062, 0.097
Fitness group classes	203.415 *	26	0.922	0.909	0.060	0.074	0.062, 0.097
<6 months experience	182.192 *	26	0.927	0.901	0.058	0.074	0.061, 0.098
≥6 months experience	227.677 *	26	0.928	0.902	0.059	0.076	0.068, 0.098

**Note:** χ^2^ = chi-square test; df = degrees of freedom; CFI = comparative fit index; TLI = Tucker–Lewis index; SRMR = standardized root mean square residual; RMSEA = root mean squared error of approximation; 90% CI = 90% confidence interval of RMSEA. * = *p* < 0.001; DNC = did not converge.

**Table 2:** Factor loadings and reliability coefficients of the two-correlated-factors ESEM.

Item	λ Preference	λ Tolerance
Preference	*0.83*	
Item 2	0.652 **	0.175 *
Item 4	0.679 **	0.254 **
Item 5	0.785 **	0.189 **
Item 6	0.454 **	0.539 **
Item 7	0.633 **	0.177 **
Tolerance		*0.78*
Item 1	−0.011	0.721 **
Item 3	0.191 **	0.637 **
Item 8	0.214 **	0.761 **
Item 9	0.421 **	0.617 **
Item 10	0.212 **	0.512 **

**Note:** λ = standardized factor loading; target loadings are in bold; composite reliability coefficients are in italics; ** = *p* < 0.001; * = *p* < 0.05.

**Table 3:** Multigroup analysis of the ESEM specification.

Model	χ^2^	df	CFI	ΔCFI	TLI	ΔTLI	SRMR	ΔSRMR	RMSEA	ΔRMSEA
Exercise Type										
Configural	239.379 *	52	0.940	-	0.906	-	0.038	-	0.081	-
Weak	270.181 *	68	0.935	0.005	0.914	0.008	0.048	0.010	0.074	0.004
Strong	290.488 *	76	0.931	0.009	0.916	0.010	0.049	0.011	0.072	0.009
Strict	311.708 *	86	0.927	0.013	0.924	0.018	0.059	0.021	0.069	0.012
Exercise Experience										
Configural	133.720 *	52	0.906	-	0.889	-	0.048	-	0.096	-
Weak	151.286 *	68	0.904	0.002	0.883	0.006	0.060	0.012	0.085	0.011
Strong	163.104 *	76	0.900	0.006	0.881	0.008	0.062	0.014	0.082	0.014
Strict	180.087 *	86	0.899	0.007	0.887	0.002	0.073	0.025	0.080	0.016

**Note:** χ^2^ = chi-square test; df = degrees of freedom CFI = comparative fit index; TLI = Tucker–Lewis index; SRMR = standardized root mean square residual; RMSEA = root mean squared error of approximation; Δ = difference; * = *p* < 0.001.

## Data Availability

Under licensing, the data were exclusively used for the current study. The data used to support the conclusions are not publicly available, but they can be obtained with a reasonable request and the permission of the Life Quality Research Center and the corresponding author.

## Supplementary Materials

The following supporting information can be downloaded at: https://www.mdpi.com/article/10.3390/ejihpe13070086/s1.
