# Supplementary material for: Using Psychometric Testing Procedures for Scale Validity, Reliability, and Invariance Analysis: The PRETIE-Q Portuguese Version
Source: Eur J Investig Health Psychol Educ. 2023 Jun 23;13(7):1158–72. doi: 10.3390/ejihpe13070086 (PMC10378368; doi:10.3390/ejihpe13070086)
Supplement: Supplementary file 1 [file ejihpe-13-00086-s001.zip › ejihpe-2408746-SI.pdf]

Supplemental Material

Mplus syntaxes.

```
USEVARIABLES ARE
pt1 pt2 pt3 pt4 pt5
pt6 pt7 pt8 pt9 pt10;
```

Unidimensional (Model 1)

```
ANALYSIS:
ESTIMATOR IS MLR;

MODEL:
UNI by pt1 pt2 pt3 pt4 pt5
pt6 pt7 pt8 pt9 pt10;

OUTPUT:
STDYX.
```

Correlated two-factor CFA (Model 2)

```
ANALYSIS:
ESTIMATOR IS MLR;

MODEL:
TOL by pt1 pt3 pt8 pt9 pt10;
PREF by pt2 pt4 pt5 pt6 pt7;

OUTPUT:
STDYX.
```

Correlated two-factor ESEM (Model 3)

```
ANALYSIS:
ESTIMATOR IS MLR;
ROTATION IS TARGET (OBLIQUE);

MODEL:
PREF by pt1-pt10
pt1~0 pt3~0 pt8~0 pt9~0 pt10~0(*1);
TOL by pt1-pt10
```

```
pt2~0 pt4~0 pt5~0 pt6~0 pt7~0(*1);

OUTPUT:
STDYX.
```

One bifactor and two-correlated CFA (Model 4)

```
ANALYSIS:
ESTIMATOR=MLR;

MODEL:
G by PT1-PT10;
TOL by pt1 pt3 pt8 pt9 pt10;
PREF by pt2 pt4 pt5 pt6 pt7;

OUTPUT:
STDYX.
```

One bifactor and two-correlated ESEM (Model 5)

```
ANALYSIS:
ESTIMATOR=MLR;
ROTATION=TARGET (orthogonal);

MODEL:
G by
PT1-PT10 (*1);
PREF by pt1-pt10
pt1~0 pt3~0 pt8~0 pt9~0 pt10~0(*1);
TOL by pt1-pt10
pt2~0 pt4~0 pt5~0 pt6~0 pt7~0(*1);

OUTPUT:
STDYX.
```
